# BioCarian: search engine for exploratory searches in heterogeneous biological databases

**DOI:** 10.1186/s12859-017-1840-4

**Published:** 2017-10-02

**Authors:** Nazar Zaki, Chandana Tennakoon

**Affiliations:** Department of Comp. Science and Software Engineering, College of Info. Technology, United Arab Emirates University (UAEU), Al Ain, PO Box 15551 United Arab Emirates

**Keywords:** Search engine, Exploratory search, Biological databases, Heterogeneous databases, RDF, SPARQL

## Abstract

**Background:**

There are a large number of biological databases publicly available for scientists in the web. Also, there are many private databases generated in the course of research projects. These databases are in a wide variety of formats. Web standards have evolved in the recent times and semantic web technologies are now available to interconnect diverse and heterogeneous sources of data. Therefore, integration and querying of biological databases can be facilitated by techniques used in semantic web. Heterogeneous databases can be converted into Resource Description Format (RDF) and queried using SPARQL language. Searching for exact queries in these databases is trivial. However, exploratory searches need customized solutions, especially when multiple databases are involved. This process is cumbersome and time consuming for those without a sufficient background in computer science. In this context, a search engine facilitating exploratory searches of databases would be of great help to the scientific community.

**Results:**

We present BioCarian, an efficient and user-friendly search engine for performing exploratory searches on biological databases. The search engine is an interface for SPARQL queries over RDF databases. We note that many of the databases can be converted to tabular form. We first convert the tabular databases to RDF. The search engine provides a graphical interface based on facets to explore the converted databases. The facet interface is more advanced than conventional facets. It allows complex queries to be constructed, and have additional features like ranking of facet values based on several criteria, visually indicating the relevance of a facet value and presenting the most important facet values when a large number of choices are available. For the advanced users, SPARQL queries can be run directly on the databases. Using this feature, users will be able to incorporate federated searches of SPARQL endpoints. We used the search engine to do an exploratory search on previously published viral integration data and were able to deduce the main conclusions of the original publication. BioCarian is accessible via http://www.biocarian.com.

**Conclusions:**

We have developed a search engine to explore RDF databases that can be used by both novice and advanced users.

**Electronic supplementary material:**

The online version of this article (doi:10.1186/s12859-017-1840-4) contains supplementary material, which is available to authorized users.

## Background

There is a large number of biological databases that have become available in the public domain in recent years. According to the latest NAR database edition, there are more than 1600 listed database [1]. This is an under representation of the total number as there are many commercial and private databases. The number and size of private databases are in the rise [2, 3] mainly due to high throughput technologies being used in biological research. These biological databases can be in standard formats like flat files, VCF, XLS, GFF, BED etc [4, 5]. or other user defined formats. Furthermore, some databases are only accessible through an API or via a website (e.g. genecards.org).

Searches on these databases can be categorized as exact searches and exploratory searches. In exact searches user has the complete idea of what he is searching for while in exploratory searches, user only has a vague idea about what he is searching for. An example for the former type of search is a search for information on a specific gene, and an example of the latter type of search is finding the answer to the question “what are the possible cancer causing genes in an experiment?”. Finding the answer to an exact search is not difficult and all major databases [6–10] have excellent interfaces for such searches. However, the question of exploratory searches of these databases is not well addressed.

To find an answer to a query, a scientist may generally need to access several databases. For example, finding a mutation relevant to a disease using the result of an NGS experiment may require searching across several databases containing information on genes, proteins and diseases. For a scientist who is not versatile in programming and IT, this type of a search may be a tedious task. Having a search engine for performing exploratory searches across several databases will be very useful for them.

Semantic web technologies have developed methods for linking diverse sources of data. As such, it provides a well-established method for integrating different databases. Semantic web methods require databases to be in Resource Description Format (RDF) format. There are several popular databases that are already in RDF format (e.g. Ensemble [7], UniProt [10], GWAS [6]) and several projects are actively converting popular databases into RDF format (e.g. [9, 11]). Nevertheless, there are many databases like those at the National Center for Biotechnology Information (NCBI) that are not accessible in RDF format. To make queries from RDF data, an SQL-like query language called SPARQL (A recursive acronym for SPARQL Protocol and RDF Query Language) has been developed [12]. Its learning curve is not very steep especially for those having a background in SQL. SPARQL is a powerful language that can query multiple databases. Through its federated search capabilities, SPARQL can even run queries on databases that are hosted by different institutions. Furthermore, SPARQL can be integrated with full-text searches. SPARQL can be very useful in database searches due to these features.

There are many methods used to access semantic databases. A common method is to provide an interface to write direct SPARQL queries. The interface may simply be a text box to write queries or may contain some additional features (for example, enumeration of available values for query construction and query templates that users can customize). There are query builders that construct SPARQL queries graphically [13–17]. These constructors may support federated queries [17] and the construction methods range from building a query from scratch to assembling elements from pre-defined structures of the database [15]. Another technique is to explore the databases using graphs that show the connections between the elements in the databases [16].

An advantage of direct SPARQL querying is that the full power of SPARQL can be unleashed. However, for users without any knowledge in SPARQL this type of interface is not valuable. The graphical query builders may be suitable for constructing simple queries, but advanced query construction is not possible with these builders as they support only a limited set of commands, and the user interface becoming convoluted when many entities are involved in a query. Users may find that investing time to learn the basics of SPARQL to be better than spending time on constructing queries using the builders.

Some direct SPARQL based interfaces provide the ability to do free-text search, but some do not have free-text search integrated. Query constructors evaluated here do not provide free-text search capabilities. Several graph based solutions and facet based solutions have free-text search capabilities. However none of the indirect querying methods had the capability to initiate a search with a SPARQL query.

When performing exploratory searches, the user starts with a broad idea in mind and starts to modify his/her search based on the results presented in previous searches. Therefore, it is essential that the user be provided with information that can help guide his/her search. A common way of providing such information is via facets. Facets provide a list of categories and available choices for each category in the search result. They help users narrow down the search space. Faceted navigation will also have issues when the number of facets and facet values become large. They would be problematic to display and if a facet contains hundreds of facet values, it will be hard to navigate. Existing faceting systems use ranking by frequency and displaying an arbitrary number of facet values to handle such cases. These methods do not completely address the issue, and we need to find better solutions. It would be valuable if the display of facets can be constructed in a way that can cut through clutter and help users get an idea about the relevance of each facet value.

Among the methods presented, facets are the most intuitive and familiar approach for an average user, since anyone familiar with browsing the internet is bound to have come across faceted navigation in many forms. In the context of exploratory searches writing direct SPARQL queries and using query constructors is not a practical solution as such an approach will need the creation of new queries in each iteration of the search.

We will survey some semantic web browsing solutions that incorporate facets. Openlink Virtuoso’s [18] faceted search is a popular facet interface used by many projects like Bio2RDF [11] and DisGeNet [8]. It can start with a free text search and provides a basic faceting service. As it is a general faceted browser, the descriptions of facets and facet values are taken directly from the RDF database. These descriptions can be cryptic. Compared to this, Linked Life Data [9] provides a modern faceting system that is user friendly. Apart from these traditional faceting methods there are several other methods that have been developed. These are not practically used in large scale biological databases. mSpace [19] is a system where facets are organized in a changeable hierarchy and selecting a facet value high up in the hierarchy will affect the selection of the facets lower in the hierarchy. Longwell [20] is a tool in the Simile project that can be deployed in a generic RDF dataset to create a faceted search engine uses the display vocabulary Fresnel [21] for reporting the results. /facet [22] is a faceted browser that can generate facets automatically on heterogeneous linked data when ontological information about the dataset is available. Parallax [23] is a faceted browsing concept that uses facets to browse connected sets. Humboldt [24] and Tabulator [25] are two more faceted browsers that allow switching between different sets of facets. In gFacet [26] facets are represented as nodes in a graph where arcs depict the dependencies of the facets. Faceting methods generally show facets directly connected to the query [19, 20, 27] while some can filter using facets that are not directly connected [23–25, 28]. Some methods show the complete facet hierarchy [25, 28] while in others [23, 24, 27] the hierarchy is not completely visible.

We observe that most biological databases are stored in structured file formats (or they can be accessed in a structured format like JSON or XML) and they can be converted in to tabular formats. There are existing methods for converting tabular data into RDF format [29–33] (W3C recommendations can be found at www.w3.org/TR/csv2rdf). Some try to automate the conversion process [34, 35] and others like Google Refine takes a semi-automated approach. There are converters targeting fixed data sets (e.g. NCBI2RDF [36] providing an RDF interface to NCBI data) and more general methods like D2R [37] designed to map relational database schemas into OWL and RDF vocabularies.

In this paper we present BioCarian, a search engine for exploring biological databases utilizing semantic web methods. We start by converting tabular data into RDF format. This conversion not only turns tabular data to RDF, but also generates some additional information that helps in building a faceted search engine. The search engine provides an interface where SPARQL queries can be run on the converted RDF database. A free-text search option and a user friendly editor is provided to enter SPARQL queries. For those users who do not know the SPARQL language, an enhanced faceted interface to explore the databases is provided. The facet interface has several ranking methods to identify most relevant facet values in a given context. These methods can guide users in locating a narrow set of facet values when a large number of choices are presented. The facet interface can also be used to create advanced SPARQL queries. Furthermore, the search engine integrates the facet interface with free-text and custom SPARQL queries.

## Implementation

BioCarian requires an RDF database with a specific structure to operate on. This database can be the union of several different databases. The original databases maybe in various formats like flat files, variant call format(VCF), excel(XLS), general feature format(GFF), browser extensible data(BED) or RDF. However, all of these can be converted to tabular data. (The instructions and tools for converting popular file types to tabular data are provided in the BioCarian website.) The search engine requires the knowledge of the database structure to properly display search results and facets. This structure is defined using Resource Description Framework Schema (RDFS) (https://www.w3.org/TR/rdf-schema/). For this discussion, we will assume that the databases are already in tabular form.

### Design of the database

A table can be thought of as a collection of objects where each row is a subject and the columns are predicates. With this abstraction, each cell in the table can be represented as a subject-predicate-object triplet in RDF. Each database is assigned a unique namespace. The *i* th row will be given the subject name *N*:*i*, where *N* is the namespace of the database. The *j* th column of the table will be given a descriptive predicate name, *N*:*P*_*j*_. The cell (*i*,*j*) will be an object. The basic goal of the search engine is to find row subjects matching the search criteria and displaying the data related to those subjects. Facets for a search result are generated by enumerating predicates corresponding to row subjects in the result, and facet values are the enumeration of corresponding objects of the predicates.

As an example, consider a table containing data from the dbSNP database. It can be assigned the namespace www.dbsnp.com. It may have a column with the name SNP_Name. Suppose the 100th row contains the value rs17216163 as the SNP_Name. This can be modeled as the (subject, predicate, object) 3-tuple (www.dbsnp.com\100, www.dbsnp.com\SNP _Name, rs17213)

**Fig. 1:**
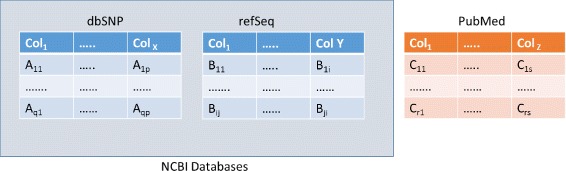
Example of a collection of databases which includes dbSNP and refSeq (from the NCBI), and PubMed (independent database)



The search engine is presented with a collection of databases in general. Each database is assigned a special rdf:type called “Database”. Some databases maybe contained inside other databases. For example, dbSNP and refSeq databases are contained inside the NCBI database collection. The databases are modeled using rdfs:Class and rdfs:subClass resources. Each database is defined as having rdf:type of rdfs:Class. If the database is inside the class *C* then it is considered to be an rdfs:subClass of *C*. Consider the example of Fig. 1, where dbSNP and refSeq are from the NCBI database collection, and PubMed is another independent database. The name of each database should be unique. We can model these as

The search engine will determine the available databases and display the search results separated by the database.

#### Database structure

The columns of a tabular database corresponds to predicates. The rdfs:domain resource is used to describe this relationship between a database and a predicate. If predicate *P* is from a column in database *D*, we express this by the tuple.

P rdfs:domain D

There are predicates that are not independent of each other. For example, the chromosome and the location of a Single Nuecleotide Polymorphism(SNP) might be recorded as two column entries in a table. However, displaying the location by itself is meaningless without any knowledge of a chromosome value. Furthermore, independently selecting facet values from dependent facets can lead to the formation of bad queries. In such cases, the contents of one facet must be updated depending on the choices in the other facet. Two facets *F*1 and *F*2 that are not independent are indicated by the resource rdfs:seeAlso. i.e. we can write

F1 rdfs:seeAlso F2

or

F2 rdfs:seeAlso F1

When facet values are generated, the dependent facet value is added as a prefix separated by a colon.

As an example, consider a table of SNPs that contain two columns indicating the chromosome and genomic co-ordinates of a SNP. Although they are independently stored, genomic co-ordinate will be meaningless if shown by itself as it will be just a set of numbers without any context (for example, there may be several SNPS having the same genomic co-ordinate in different chromosomes and the user will have no idea which is which). However, if we add the chromosome separated by a colon as a prefix to the genomic co-ordinate, it will provide the required context.

Additional attributes for the database can be specified. In the dbSNP table previously described, we gave the predicate the short name SNP_Name that is not very descriptive. Rather than this name, we can assign a more human-readable name such as Name of the SNP to be displayed by the search engine. In the database some facet values are not very useful for the user. For example some facet values might be constant or unrelated (like the bin numbers in the genome browser tables). These facets can be marked as hidden and the browser will not generate facets for them unless the user specifically issues a command. It is not necessary to index facets like the strand or p-values for free-text search. The former will result in noisy hits and the latter is unlikely to be free-text searched. Such facets can be marked as not to be indexed. We can also specify the data type of objects and the order a given predicate and its value are shown in the result screen. These facet related properties are described as RDF statements about corresponding predicates.

The user can either write the database structure by hand or a script is included that will create the structure from a configuration file. The vocabulary adapted by Biocarian to describe the structure of databases is less complex than approaches like D2R. It assumes that the table conversion has already been done and so does not require the specifications needed to run the conversion like D2R. Compared to other methods, converting the database schema is only a part of the conversion process. Biocarian needs to add extra information that will facilitate the display and generation of facets, as well as the display of search results. i.e. Biocarian describes the structure of a database to be useful for a faceted search engine in a way similar to Fresnel [21] describing how RDF entries are to be displayed.

#### Design of the search engine

The search engine can perform free-text, SPARQL based or facet based searches. Faceted searches can be combined with both free-text and SPARQL bases searches. If the user starts with a free-text search, the results of the query along with related facets are displayed. In a SPARQL based search, user uses an editor to write SPARQL queries. All the available facets are shown if the user prefers a faceted search.

**Fig. 2:**
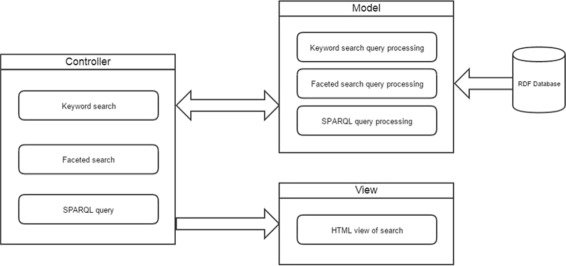
Design of BioCarian: Biocarian is designed using an MVC model. The controller accepts queries and the model interacts with the RDF database, while the view is responsible for the final display of the web pages

The search engine uses a model, view, controller design. Figure 2 shows the outline of Biocarian’s operation. The controller processes the user query entered via a free-text search box, an editor for SPARQL or facets. The models interact with the RDF database. They convert the queries gathered from the controller into SPARQL queries, sends them to a specified SPARQL endpoint and receives the query results. The views display the query interface and updates the user interface by displaying the search results and facets.

For free-text and SPARQL based queries, the facets are generated based on the search result. The results and facets are arranged by the database. For free-text searches, a score that reflects the quality of text match and a star rating that shows the relevance among the search results is displayed. The user can explore the databases he/she chooses by selecting facets. Complex queries can be built by using conjunction and disjunction of facet values.

The search engine is targeted at biological databases. When it encounters ID’s for genes, proteins, SNPs, pathways and publications, hyperlinks to find additional data on these entities is provided. Furthermore, the design of facets is done aiming to accomplish common tasks in biological research. Typically, users exploring biological databases are interested in the average or extreme facet values or in searching for specific facet values. For example, users are interested in genes that are appear with a normal, high or low frequency or might want to know if a specific gene is available. The facet values are color coded with grading to show how far each value is from the average. This will enable users to get a visual impression of the facet value distribution at a glance. Users can select, then zoom in and out of extreme and average values in facets. When there is a large number of choices available for facet values, the number of choices can be reduced by limiting them to what the user wishes to investigate. Users can also free-text search for specific facet values. If a facet value has a high frequency in the database, it has a high chance of appearing in search results just by chance. Users might like to avoid such cases and concentrate on results that are more specific to his query. We have designed our facet navigation to cater these types of common searches.

For free-text queries, a reverse-text index constructed using Apache Lucene is used together with SPARQL. Lucene is used to create the reverse index for free-text search. We make use of the built-in support Jena provides for Lucene. When constructing the free-text index, values allowed to be free-text searched are indexed with the subject as the key. We use StandardAnalyzer as the default text analyzer, however this can be changed by the user. The index is built using the default index builder. It indexes plain literals and stores the complete literal. Only the literals corresponding to user-specified properties are indexed. If there is a free-text match by Lucene, the corresponding subject in the RDF database will be returned. The storage of RDF is done using the TDB component of Jena with default settings.

#### Searching with SPARQL

The search engine generates a SPARQL query that returns all the subjects in the database matching the search criteria specified by the user interface. The search criteria can be a free-text or SPARQL query together with a facet value selection. If free-text is entered, it is translate into a SPARQL query that searches the Lucene index and returns matching subjects with the match score. If a SPARQL query is entered, it must be written so that a list of row subjects are returned. The following algorithm shows how the facets and facet values are generated.



For a free text search only the best matches (default value = 300) that do not score below a percentage of the top score (default 25%) are retained. The subjects are sorted according to the match score so that the most relevant hits appear first. If there are more than 300 hits, user is given the option to see more results.

#### Conversion of queries to SPARQL

We will now describe the process for converting queries into SPARQL. For each type of query (free-text, SPARQL or faceted) there is a templated query called the *K* *e* *y*_*Q* *u* *e* *r* *y*. For a simple free-text query, this will have the form



where Search_Limit is the number of best matches to retrieve from the text index. If facets are used to add additional conditions, the *K* *e* *y*_*Q* *u* *e* *r* *y* will have additional restrictions. For example the query,



will add to the previous query entries having facet *P* *R* *E* *D*1 containing values *V*1 or *V*2 and restricted to the facet values *V*3 and *V*4 from the facet *P* *R* *E* *D*2. The full algorithm for constructing the *K* *e* *y*_*Q* *u* *e* *r* *y* using different templates is given in the Supplementary (Additional file 1).

Once the *K* *e* *y*_*Q* *u* *e* *r* *y* has been constructed, information necessary for facet generation can be gathered using the following query:



Here, *Seperator* is some special string. This query will return a set of 2-ples of the form (?*f* *a* *c* *e* *t* *n* *a* *m* *e*,?*t* *o* *t* *a* *l*). In these 2-ples, ?*f* *a* *c* *e* *t* *n* *a* *m* *e* will have a facet and a facet value separated by the special string *Seperator*, and ?*t* *o* *t* *a* *l* will be the frequency of that facet value in the query result.

#### Displaying query results

Executing *K* *e* *y*_*Q* *u* *e* *r* *y* will return a set of values corresponding to the variable ?*s* *u* *b* *j* *e* *c* *t*. For free-text queries each ?*s* *u* *b* *j* *e* *c* *t* will have a score ?*s* *c* *o* *r* *e* associated with them. The variable ?*s* *u* *b* *j* *e* *c* *t* collects all the subjects that match the search criteria. All the predicates and objects related to these subjects can be retrieved by the query



The ?*s* *u* *b* *j* *e* *c* *t* *s* will be sorted by ?*s* *c* *o* *r* *e* in case of a free-text search, and will be separated by the databases they belong to. If a predicate is not marked to be displayed in the database specification, it is discarded. Other predicates are sorted by the display order stated in the database specification and the user-friendly name is displayed along with the corresponding object. If the object has a known type it is formatted with additional information (e.g. clickable link or a clickable button providing additional information about the object).

#### Facet value generation for exploratory searches

Let us assume that a database contains *N* distinct facet values for a given facet, labeled *n*_1_,…,*n*_*N*_ and there are *c*_1_,*c*_2_,…,*c*_*N*_ entries in each category respectively. Assume that there are $c_{1}^{'},c_{2}^{'},\ldots,c_{N}^{'}$ entries respectively in each category after a query. In cases where the user might want to know some property that has the highest/lowest representation, we can rank facet entries by the descending/ascending order of their frequency *c*_1_,*c*_2_,…,*c*_*N*_.

If the user is browsing a facet that is ordered by the frequency of facet values, the average values can be displayed by reporting the facet values having frequency in the interval (*μ*−*M* *σ*,*μ*+*M* *σ*), where *M* is some positive number and *μ* is the mean and *σ* is the standard deviation of the facet value frequencies. By decreasing *M*, values that are closer to the average can be found. For finding values in the upper (lower) extremes, frequencies that are larger (lower) than $\mu +\bar {M}\sigma (\mu -\bar {M}\sigma)$ can be filtered for some positive integer $\bar {M}$. By changing the value of $\bar {M}$, the values close to the average can be zoomed in and out.

**Fig. 3:**
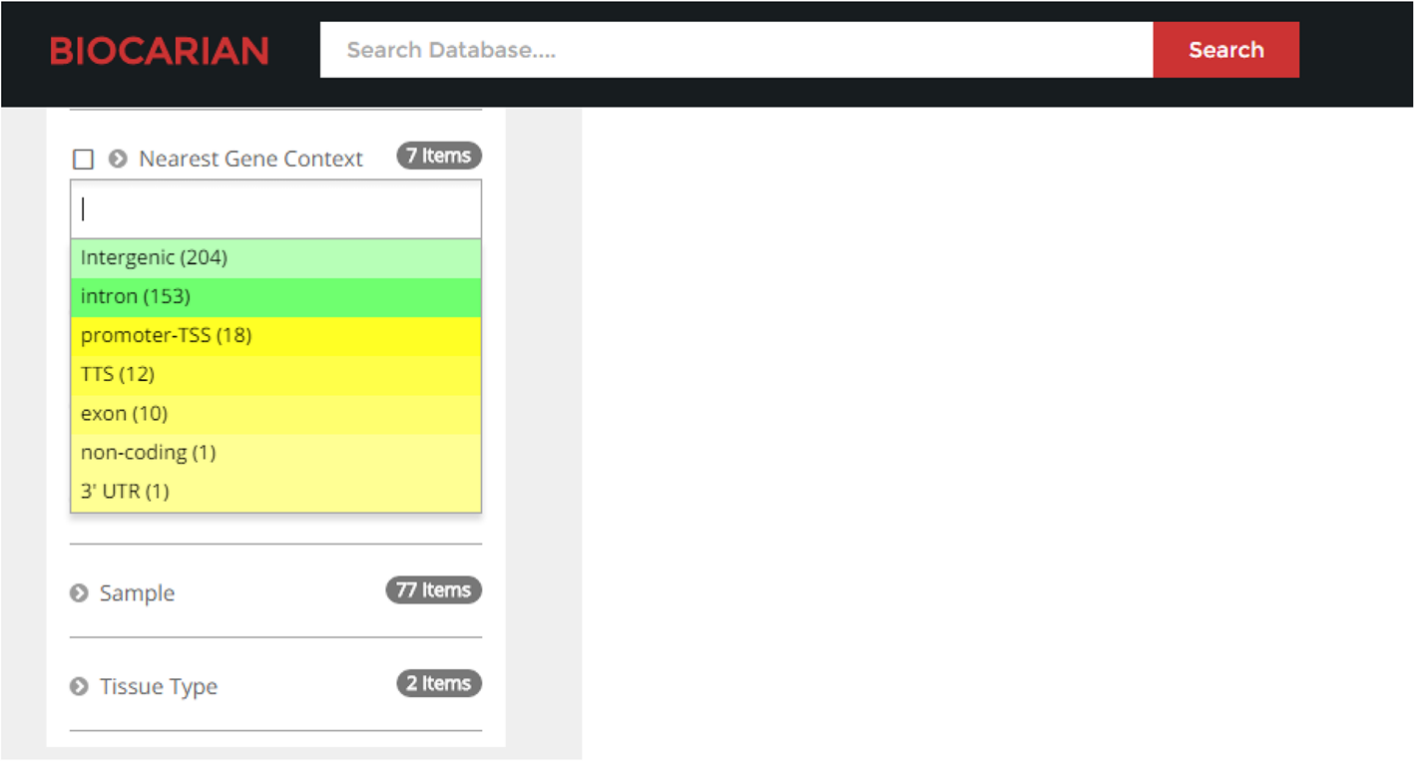
Facet display with color gradient showing the extremeness of facet values. Green indicates that the frequency of such a value is above average. Yellow indicates that the frequency of facet value is much less than average. Lighter the color more extreme the deviation will be

In addition, we can give an idea about the extremeness of a facet value with frequency *f* by assigning it a color with brightness that is proportional to $\frac {f-\mu }{\sigma }$. Figure 3 shows two examples where such color gradients have been used. If (*f*−*μ*)/*σ*>0 a yellow hue has been used (i.e. facet values that have a higher frequency than average will appear with lighter shades of yellow). Otherwise, a green hue has been used (i.e. facet values that have a lower frequency than average will appear with lighter shades of green).

In some cases the frequency counts can be misleading. If a facet value is over-represented in a database, then it may appear with a high frequency in a facet simply by chance. Sometimes it is better to have an idea of how important each facet value is to the result of the query. A way to solve this problem is to find the probability of a facet value appearing by chance in any query. If this probability is low, then the facet value has a high significance for the current query.

Let us consider the facet value *n*_*i*_. We would expect an entry in this category to be selected with a probability $p_{i}=\frac {n_{i}}{\Sigma _{j=1}^{N} n_{j}}$. We can calculate the probability of selecting $n_{i}^{'}$ elements from the category *n*_*i*_ by the formula $\alpha _{i}=P(X=n_{i}^{'}|Bin(\Sigma _{j=1}^{N} n_{j}^{'},p_{i}))$. A lower value of *α*_*i*_ indicates that the category *n*_*i*_ appears with a higher or lower probability than we expect. We can rank these categories by the ascending order of *α*_*i*_. Similarly, we can rank facet values according to their over or under representation. $\beta _{i}=P(X>n_{i}^{'}|Bin(\Sigma _{j=1}^{N} n_{j}^{'},p_{i}))$ and $\gamma _{i}=P(X<n_{i}^{'}|Bin(\Sigma _{j=1}^{N} n_{j}^{'},p_{i}))$ expresses the probabilities that the category *n*_*i*_ is over or under represented in the query. When probabilities have been used to rank facet values we can use a different approach to filter relevant results. If the top probability is *P*_*M*_, we report only those facet values with the probability smaller than *λ* *P*_*M*_ for some positive *λ*. This will reject all the facet values with probability exceeding the best facet value by *λ* times or more. By changing the value of *λ* the significant values can be zoomed in and out.

#### Remote queries

Biocarian can be used to query data that is not stored locally. The first way is to point the SPARQL endpoint to a remote SPARQL endpoint (this option is under the settings menu). If the new SPARQL endpoint has the required structural information, Biocarian can function on it as if is locally hosted. Biocarian also supports federated queries through its SPARQL editor. Standard SPARQL syntax for generating federated queries can be used, and an example can be found in the predefined queries available in the SPARQL editor. This example shows how to get the gene id from a uniport ID via a federated search, using the Uniprot endpoint.

### Overview of the browser interface

**Fig. 4:**
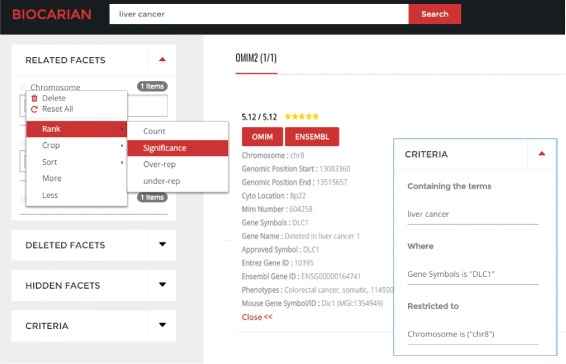
Biocarian has several features that can be used to organize facets and facet values. Facets can be deleted and activated with a context menu. The context menu also provides options to operate on facet values by ranking, filtering and sorting them. There is criteria box (shown as an inset) that shows the user the conditions of the current search

Figure 4 shows the main parts of the user interface. A search bar is provided to input free-text search. The facets are divided into three groups: related facets, deleted facets and hidden facets. related facets contain currently active facets and hidden facets contain facets that are not generally important. User can delete active facets if they are cluttering the interface, and they will appear in deleted facets. Facets in the deleted and hidden facets can be activated any time. A context menu is provided (by clicking on the chevron near the facet) and this contains the options to operate on facets and facet values. Facet values can be ranked, filtered and sorted using the context menu. Clicking the check-box near a facet will activate a conjunctive search for that facet.

To keep track of the current search, a criteria box is provided. This give a user friendly description of the current search state. If there are known biological entities (in this figure an OMIM ID and an Ensemble ID are given) clickable buttons will be generated to provide additional information from databases related to them. For free-text searches, a score and a star rating will be displayed to show the absolute and the relative relevance of the text match.

## Results

We used our framework to construct a search engine that browses several selected public databases. The databases represent a sample collection of DNA-level data (dbSNP, GWAS, Ensembl), Protein data (UniProt), pathway data (KEGG, Reactome), and disease data (OMIM, DisGeNET) and contain more than 1.4 million 3-tuples. A private database has also been added that contains viral integration sites in the liver cancer patients identified in the paper [38]. Sung et. al conducted a WGS study on liver tissue samples taken from 81 HCC patients. The samples were taken from tumors and adjacent normal liver tissue. The authors made the following observations.

1. HBV integration is more frequent in the tumors compared to normal tissues. Furthermore, integrations were present in 76 of 88 samples (≈86.4*%*) examined and are relatively frequent.
2. Recurrent integration events (where an integration is considered to be recurrent if it appears in at least 4 samples) in the genes TERT, MLL4 and CCNE1 were observed in tumor samples and account for 31 of 76 (≈40.8*%*) of the tumor samples with HBV integration.
3. HBV integrations at gene SENP5 was discovered in three samples.
4. Most integrations were near the coding genes in 209 of 399 (≈52.4*%*).
5. Among the samples having HBV breakpoints in both tumor and normal tissues, only in sample 262 there was one break-point shared between the tumor and non-tumor samples, indicating that HBV integration patterns differ in the tumor and normal samples.
6. Most of the HBV breakpoints in tumor samples were located in known coding genes, and were significantly over-represented in exon and promoter regions. In the HBV breakpoints in non-tumor samples that were located close to genes, breakpoints were mainly found in introns.
7. Only two common genes affecting both normal and tumor tissues were found, and they affected different individuals through integrating to HRSP12 (in samples 272T and 276N) and INPP4B (in samples 70T and 98N).
8. Approximately 40% of breakpoints observed were restricted to where the viral enhancer, X gene and core gene are located.

**Fig. 5:**
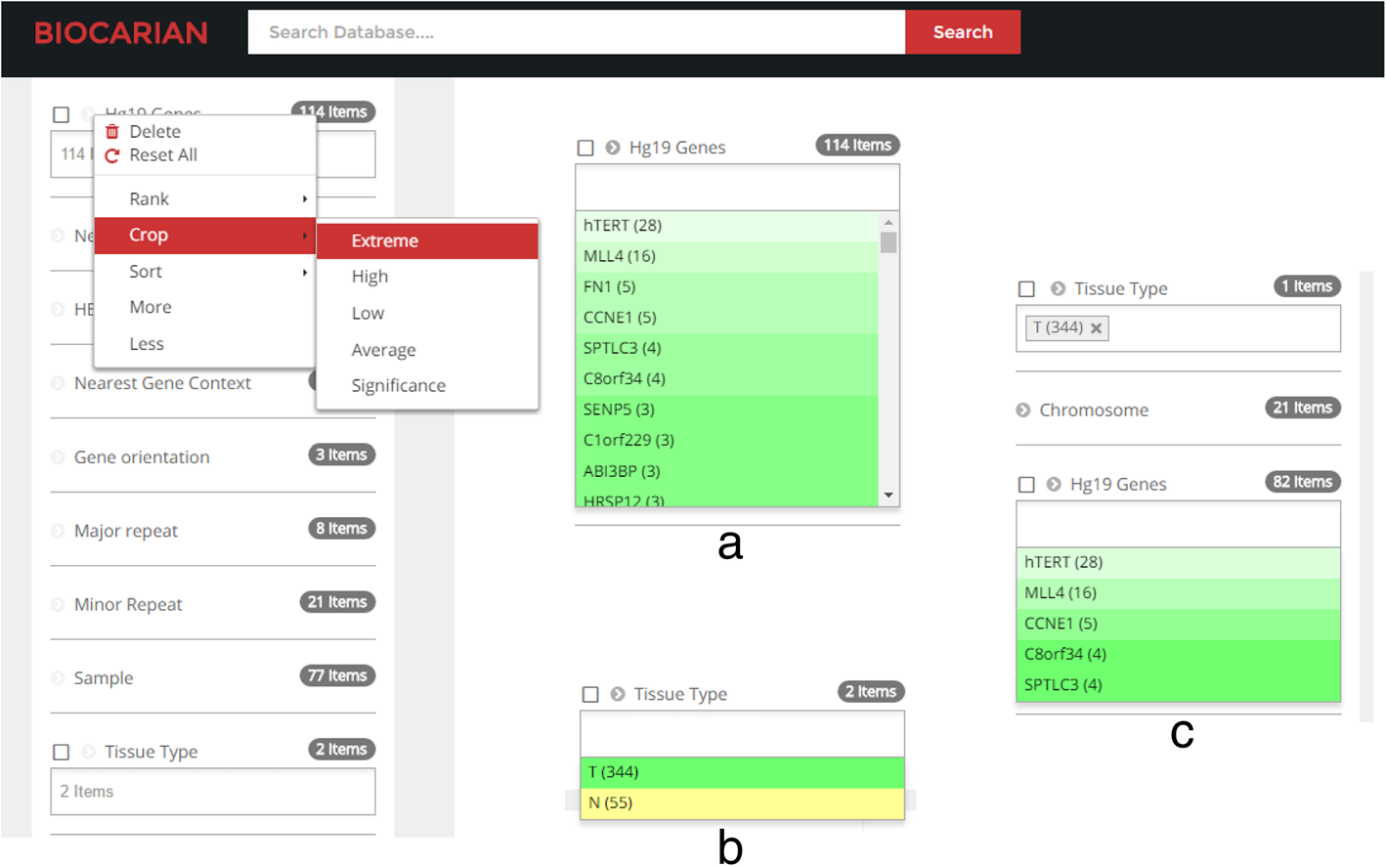
Illustration of advanced exploration of genes related to HBV integration. Our goal in this case is to find recurrent infections of genes in tumor samples where at least 4 integrations have been reported. As we can see, the initial set of genes retrieved is quite large (**a**). Therefore, we use BioCarian context menu to retrieve the recurrent integrations (**b**). To get a narrower set of genes, we get the extreme valued genes from the context menu. Initially it shows the two genes with most extreme frequencies, and selecting “More” option from the context menu shows only 5 genes that have at least 4 integrations (**c**)

In this section, we will describe how BioCarian can be used to explore this dataset and generate these observations. From the browser we can see that 77 samples out of 88 contain integrations (a percentage of 87.5%), and there are more integrations in the tumor samples (344) compared to the normal samples (55) (Fig. 5 a). The original paper reports 76 samples, but the list of integration provided actually shows 77 samples, as correctly reported by the browser.

We will next search for the recurrent integrations (i.e. integrations in genes that appear at least 4 times in the samples). There are 114 genes present in the database (Fig. 5 b). This is a large number to process.

We first study the recurrence in tumor samples by selecting only the tumor samples. There are still 82 genes available. To get a narrower set of genes, we get the extreme valued genes from the context menu. Initially it shows the two genes with most extreme frequencies, and selecting “More” option from the context menu shows 5 genes that have at least 4 integrations (Fig. 5 c).

**Fig. 6:**
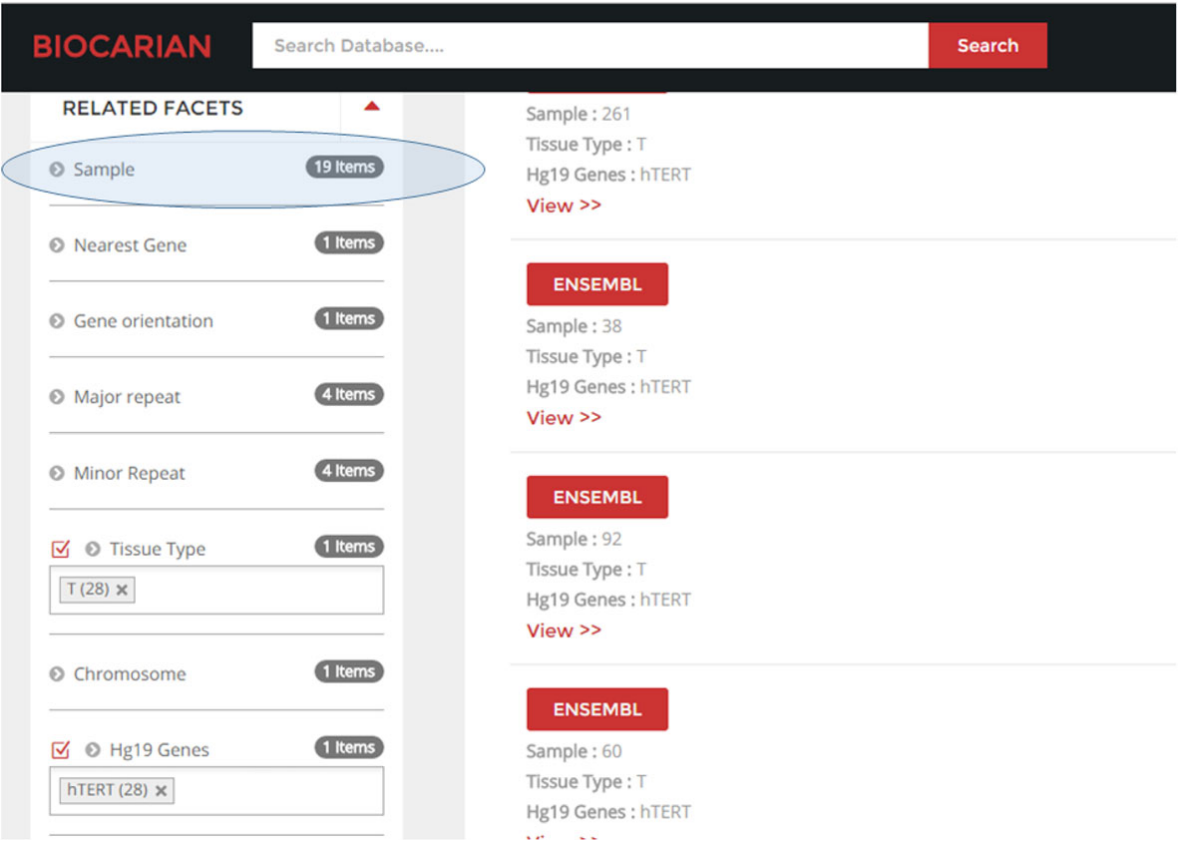
Exploring each of the candidate genes for recurring integrations shows the actual number of distinct samples integrations appear in. Here we specifically select hTERT gene, and can directly see it appears in 19 distinct samples and is thus a recurrent integration

We can see all the integrations mentioned in the paper. The color of the facet values becomes lighter as their frequency deviates more from the mean of the frequencies. For example, we can see that hTERT and MLL4 have much higher frequencies than expected in the tumor samples. When we study the hTERT, MLL4 and CCNE1 genes mentioned in the paper, we see that they have a high number of integrations, suggesting possible recurrence. However we need to see the samples they appear in to determine whether they appear in at least four separate samples. We see that they recur in 19,9 and 4 samples respectively. Other samples do not meet the stated criteria for recurrence. Then integration of C8orf34 and SPTL3C appear only in samples 71 and 23 respectively (Fig. 6). In summary the integrations appear in 42.1% (32/77) samples.

**Fig. 7:**
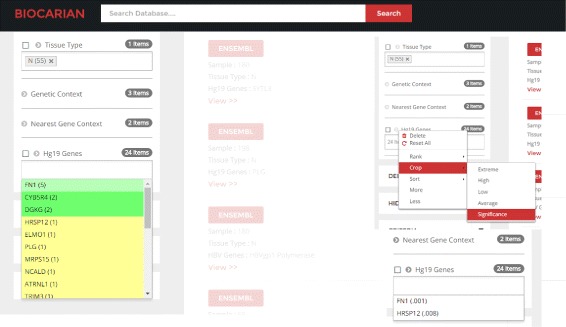
Exploring genes related to HBV integration. Our goal is to find recurrent infections of genes in normal samples where at least 4 integrations have been reported. We can see that there is only one candidate satisfying this criteria, FN1. However, we use the context menu to see which genes are significant, and only two genes are returned

When Normal tissues are examined for recurring integrations by looking at the number of integrations, we see that there is only one candidate (FN1) for recurrence in more than 4 samples. In fact, if we crop the list of possible genes by significance, we are only left with only two genes, including this gene (Fig. 7). We can see that FN1 does appear in 5 distinct samples.

**Fig. 8:**
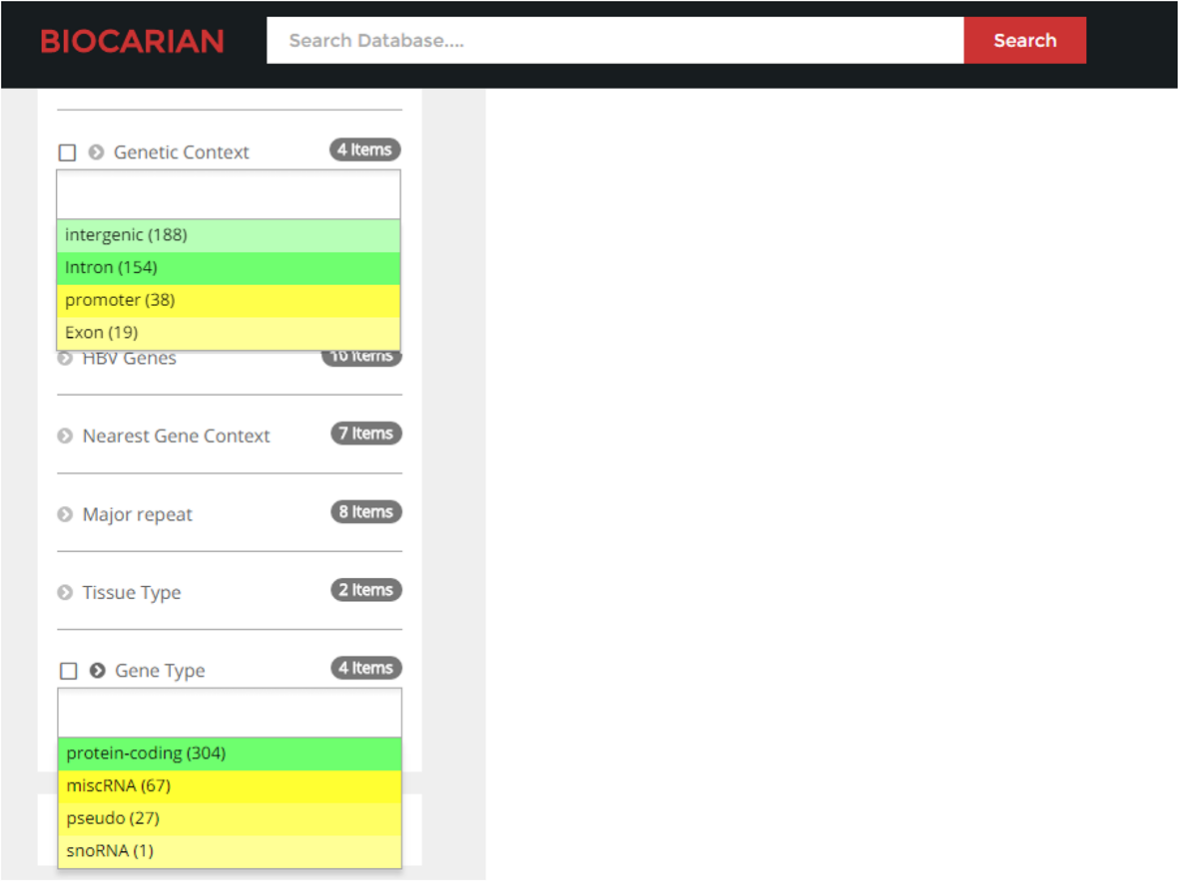
Exploring regions of HBV integration. Biocarian is being used here to see which regions show a preference to viral integration

In the regions where integrations have happened, we can see that intronic and exonic regions contain more integrations compared to intergenic regions. Since the intergenic regions are much larger than the intronic and exonic regions, we can suspect that intergenic regions are under-represented in integrations. Similar observations leads us to suspect that these breakpoints are significantly over-represented in exon and promoter regions. Similarly, we can see that most of the integrations (304 out of 399 of them) happen in protein coding genes (Fig. 8).

**Fig. 9:**
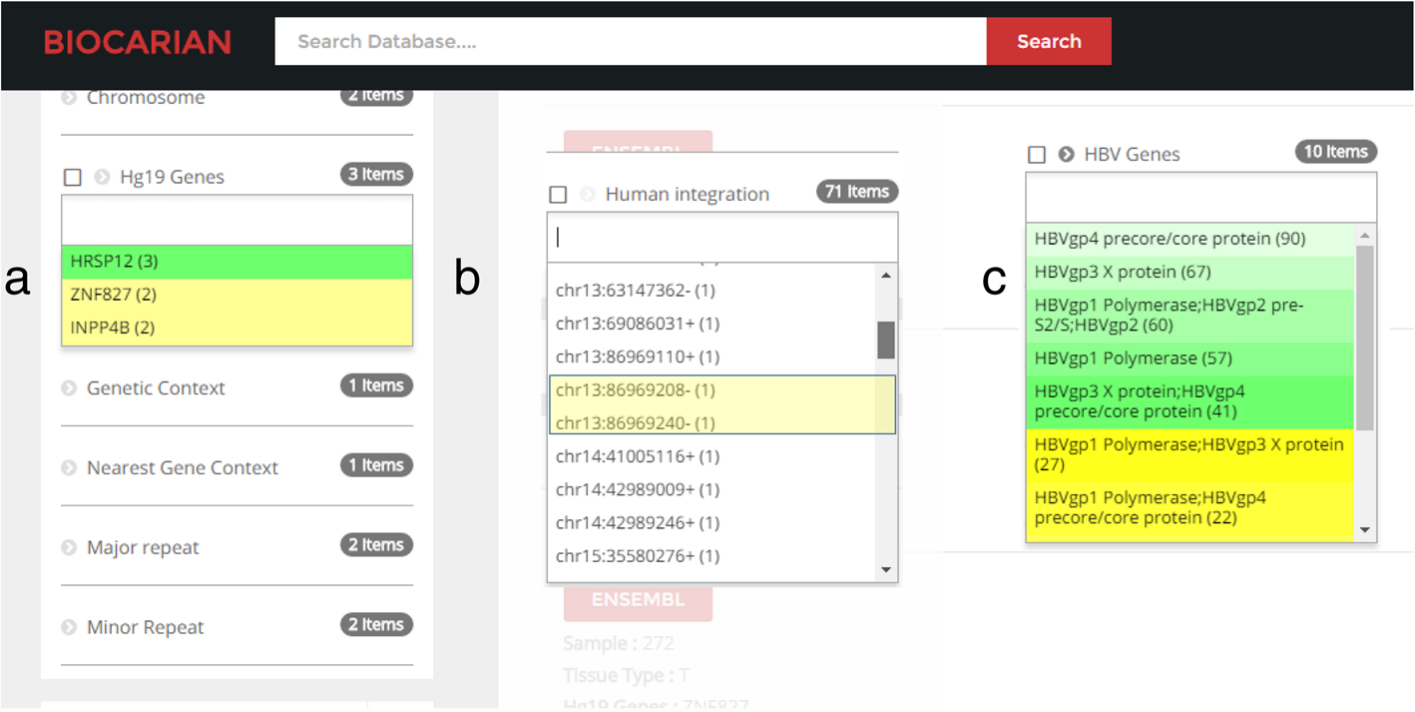
Illustration of advanced exploration of HBV integrations within normal/tumor samples. Here we use BioCarian context menu to narrow down the search by identifying the samples that contain integrations from the list of Hg19 genes (**a**), chromosomal integration of human (**b**) to explore the significant HBV integrations within normal/tumor samples (**c**)

We will next look at genes that have integrations in both normal and tumor samples. We can isolate them using a simple SPARQL query entered to the search engine. This query can be found as a template in the SPARQL editor. The resulting facets give us information that shows that three genes HRSP12, INPP4B and ZNF827 contain integrations in both the normal and the tumor samples. In fact, one of these integrations ZNF827 has been missed out in the original paper (Fig. 9 a).

We can find integrations that appear in the same sample. The simple SPARQL query given below can identify the samples that contain integrations in both normal and tumor samples, and in the same chromosome.



This produces a narrow list of 71 breakpoints. We will next sort them alphabetically and go through the list to see if there are two nearby integrations. And we see that we can find the integration mentioned in the paper (Fig. 9 b).

Finally we see how the integrations are distributed in the HBV genome. We can see that 39% of them are around the HBV protein X and Core protein regions (157 out of 399) (Fig. 9 c). We can conclude that the observations mentioned in the paper can be found using an exploratory search with our search engine.

### User survey

We conducted a survey on the usability and the usefulness of Biocarian by asking a group of 20 undergraduate biology students from the Department of Biology, College of Science, UAEU to compare it along with three other semantic-web related faceted search engines. The other chosen search engines were the linked life data search engine, Bio2RDF virtuoso faceted browser and GoPubmed. The users were asked to rank different aspects of the search engines in a scale of 1 to 5 with 1 being bad, 3 being average and 5 being excellent.

**Fig. 10:**
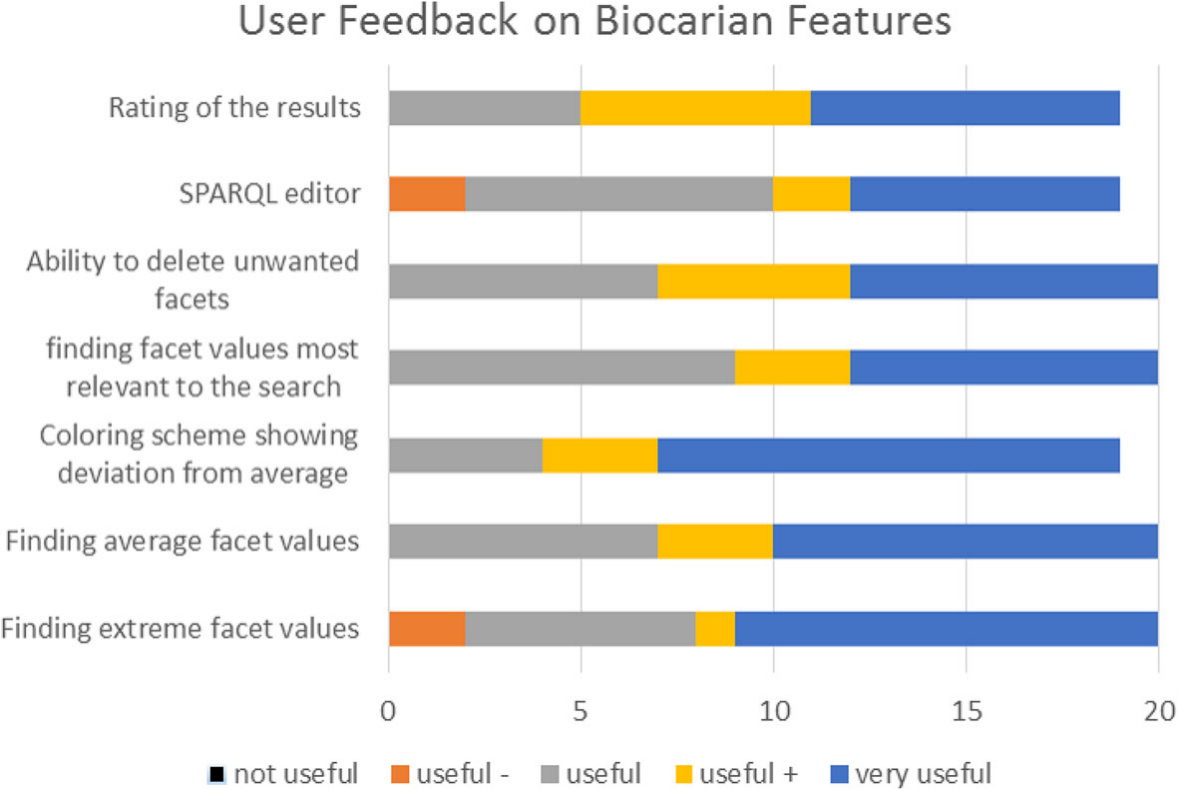
Rating of the usefulness of different features of Biocarian. Users were asked to rate the usefulness of different features of Biocarian in a scale of not useful, useful -, useful +,very useful. The figure shows the stacked graph of the responses

Figure 10 shows the results for the weighted average of the ratings. The users have rated Biocarian as the most user friendly and having the best design, with Bio2RDF and it’s HTML-browser like interface being ranked lowest in these categories. Linked life data and GoPubmed had comparable ratings. The same trend was shown in selecting the easiest search engine to navigate facets, and the methods used to organize the facets. In the category of the amount of facets shown, all the search engines were ranked almost the same with a slight edge for Biocarian over Linked life data and GoPubmed.

**Fig. 11:**
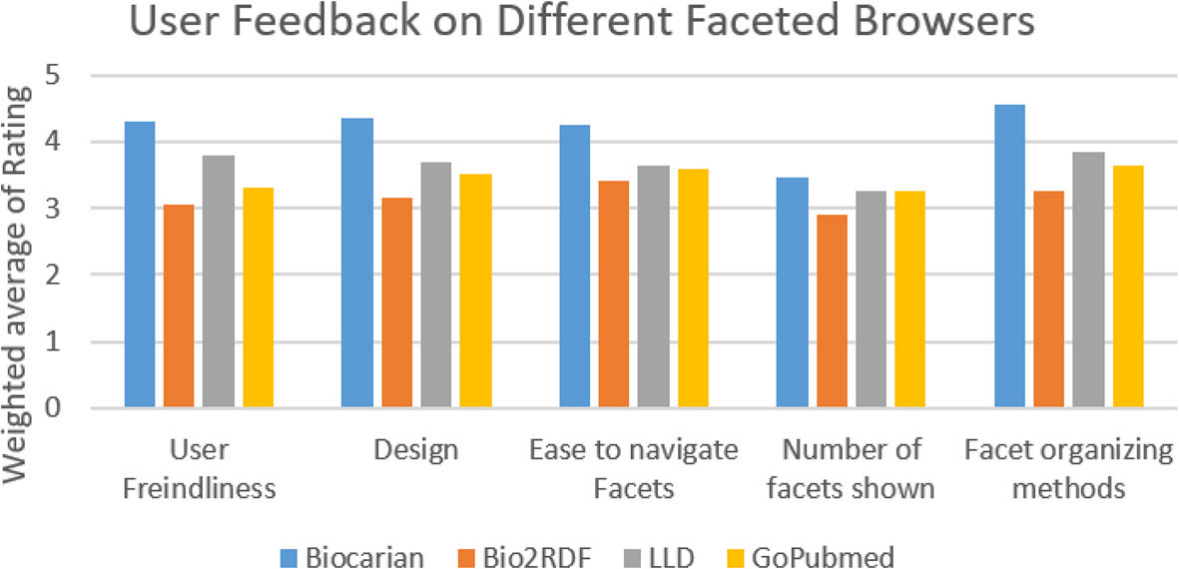
User feedback on features of different faceted browsers. Three faceted browsers for semantic web data were compared for their features, and users were asked to rate them on a scale of 1 (bad) to 5 (excellent). The figure shows the weighted average of their ratings

We then asked the users to rate the usefulness of different components of Biocarian. Figure 11 shows the stacked graph of user responses. None of the users stated that any feature of Biocarian was not useful. The ability to find the extreme values, average values and to color of the facet values according to the distance from the average were considered as very useful features by more than half the users. The users had relatively low opinions about the usefulness of finding the most relevant facet values to the search query and the usage of the SPARQL editor. In fact, only 35% of them expressed any interest in learning SPARQL. This suggests that access to direct SPARQL querying may not be essential to biologists without any IT experience. Further information on the user survey can be found in the Supplement (Additional file 2).

## Conclusion

Compared to exact searches, exploratory searches of heterogeneous biological databases is not straightforward. It requires writing of custom scripts to access and process data, and is not an easy task for a researcher without some knowledge in computer science. We provide an interface for converting and querying biological databases in RDF format. We have demonstrated that this interface can be used successfully to explore facts about HBV integration in to the human genome. Without resorting to any scripts, the facet interface along with some elementary SPARQL queries were sufficient to discover the major conclusions presented by Sung et al.

There are many faceted browsing paradigms in the literature. These methods concentrate on organizing and utilizing facets in a search but do not consider the question of locating important facet values inside a facet. Also some of these methods do not consider or cannot handle large number of facets. This is a very important problem when users have to make sense out of facets containing hundreds of choices.

We have proposed several methods that can be used by an explorer of a database to quickly narrow down what a user is searching for when facets contain many facet values (and also methods to organize facets). We have shown that in practice, these methods actually help narrow down important choices when a large number of choices are available and that with few clicks many important conclusions can be derived. We also propose that faceted search of SPARQL queries over RDF databases are a good method for exploratory searches due to their ability to perform complex queries across linked databases.

Biocarian needs to know the structure of a database to operate on it. If the content of a database changes leaving the structure intact, Biocarian can browse the database after converting the database using previously defined structure information. However, it may happen that the database schema will change and new columns may get added or deleted or their locations changed. In such cases the structure information needs to be re-generated. If it is just an addition of columns, Biocarian can still use old scheme to partially convert the database. In other case creating a new structure for the databases is unavoidable. However, unless there is a major overhaul of the database structure this task is not that difficult as parts of the structure definition can be re-used. Currently, there are attempts to describe databases in standard format that enables their conversion to RDF. Ideally, we would hope for databases to be released with structural information described in a standard format similar to Biocarian. If such information is provided with any database, Biocarian can seamlessly integrate it as the database evolves.

There are several methods proposed to integrate biological data and there are existing projects (e.g. Bio2RDF [11], Linked life data [9], KaBOB [39] and BioLOD [40]) that tackle this problem. They deal with converting, linking and storing of heterogeneous databases, and the exploration of these databases is not their major focus. In contrast, Biocarian provides a conversion scheme that is simple and concentrates more on the exploration of the converted databases. We currently provide no way of integrating two different databases based on their semantic content. For example there is currently no connection made when two different tables contain the same protein, or when these proteins are under two different names. We are currently working on a solution to connect and build a knowledge graph integrating distinct databases, based on semantic content. We make no attempt to make these connection in this version of Biocarian. We have implemented our current solution assuming warehousing of the databases. However, RDF databases with information about their structure can be hosted at different endpoints. Then, a federated database system can be implemented by modifying the existing SPARQL query conversion module.

When we analyzed the data from Sung et al. with Biocarian we were able to derive all the major conclusion. In fact, we were able to correct mistakes in their analysis where they have got a sample count wrong and missed a gene in the analysis. This shows that Biocarian can be used to do a primary analysis of data using few clicks without resorting to writing custom scripts. Also, Biocarian can act as means of an independent, orthogonal verification of an analysis result.

We therefore believe that Biocarian will be a useful tool for researchers who are not competent in computer as well as experienced bioinformaticians to explore diverse datasets.

## Availability and requirements

**Project Name:** BioCarian

**Project Homepage:** http://www.biocarian.com

**Operating systems:** Since BioCarian is a web base application, it works in all operating systems.

**Programing language:** Perl, php.

**Other requirements:** None.

**License:** Not applicable.

## Additional files

Additional file 1 SPARQL Conversion of Queries. (PDF 356 kb)

Additional file 2 Survey Detailed Results. (PDF 656 kb)

## Abbreviations

API: Application programming interface
BED: Browser extensible data
GFF General feature format; HBV: Hepatitis B virus
NAR: Nucleic acids research
NCBI: National Center for Biotechnology Information
NGS: Next-generation sequencing
RDF: Resource description format
RDFS: Resource description framework schema
SNP: Single nuecleotide polymorphism
VCF: Variant call format
XLS: Excel format
