# Supplementary material for: BioCarian: search engine for exploratory searches in heterogeneous biological databases
Source: BMC Bioinformatics. 2017 Oct 2;18:435. doi: 10.1186/s12859-017-1840-4 (PMC5625622; doi:10.1186/s12859-017-1840-4)
Supplement: Supplementary file 1 — SPARQL Conversion of Queries. (PDF 356 kb) [file 12859_2017_1840_MOESM1_ESM.pdf]

SPARQL Conversion of Queries

The basic query executed for search and facet generation is given below. For free-text, query editor or faceted search, only the content of $Key_Query variable, which holds a templated query, will change. The query returns the facets,facet values and their frequency in one single query. ?facetname contains the concatenation of each facet value with its parent facet. $seperator is a unique string that separates the facet and facet value.

```
SELECT (fn:concat(?facet,$seperator,?facetpred) AS ?facetname) (COUNT(?subject) AS ?total)
WHERE
{
    {
        $Key_Query
    }
    ?subject ?facetpred ?facet .
    FILTER($Facet_Predicates)
}
GROUPBY ?facet ?facetpred
```

Free text search

This is the template for $Key_Query when free text search is done.

```
$Key_Query=
    "
        SELECT DISTINCT ?subject ?score
        WHERE
        {
            (?subject ?score) text:query ('$Query_String' $Search_Limit).
            $Wild_Predicate
            $And_Union
            $OR
        }
    ";
```

$Query_String is the text user enters. $Search_Limit is the number of hits to fetch from the text index. Other variables are constructed as follows.

OR search

Default faceted search will let users pick a set of values from a facet, and find those results that only contain these values.Each facet value will have a format like "20543847"^^<http://www.w3.org/2001/XMLSchema#pubmed>, i.e. "facet value"^^<type>. The type may be empty for a string or may be another type (see documentation type.txt). If f_1,...,f_n are the facet values for the facet F_x, the variable $OR_Value(F_x) is created as

$OR_Value(F_x)=VALUES(?v){(f_1)(f_2)...(f_n)}

An example is

$OR_Value(GWAS:PUBMEDID)=VALUES (?v){("18846501"^^type:pubmed)("23535732"^^type:pubmed)}

A final query $OR_Union is constructed for all chosen facets F_1,F_2,...,F_m as

```
$OR_Union=
{
	$OR_Value(F_1)
	?subject F_1 ?v
}
UNION
{
	$OR_Value(F_2)
	?subject F_2 ?v
}
....
....
UNION
{
	$OR_Value(F_m)
	?subject F_m ?v
}
```

An example is

```
$OR_Union=
{
	VALUES (?v){("21")("23")}
	?subject GWAS:CHROM ?v
}
UNION
{
	VALUES (?v){("18846501"^^type:pubmed)("23535732"^^type:pubmed)}
	?subject GWAS:PUBMEDID ?v
}
```

OR search for free-text

When free text search is done, a different variable is constructed..
$OR_Value_Text(F_x)=(?p=F_x && ?o=f_1)||(?p=F_x && ?o=f_2)||......||(?p=F_x && ?o=f_n)
An example is
$OR_Value(HBV:GENE)=(?p=HBV:GENE && ?o="MLL4")||(?p=HBV:GENE && ?o="hTERT")
A final query $OR_Union_Text is constructed for all chosen facets F_1,F_2,...,F_m as
$OR_Union_Text=$OR_Value_Text(F_1)||$OR_Value_Text(F_2)..||$OR_Value_Text(F_x)
An example is
$OR_Union_Text=(?p=HBV:GENE && ?o="MLL4")||(?p=HBV:GENE && ?o="hTERT")||(?p=HBV:CONTEXT && ?o="Exon")||(?p=HBV:CONTEXT && ?o="promoter")

AND search

If multiple subjects are shown and a checkbox is selected, a conjunctive search is conducted.
If f_1,...,f_n are the facet values for the facet F_x, the variable $And_Value(F_x) is created as
$And_Value(F_x)=FILTER(?Vx IN (f_1,f_2,..,f_n))
An example is

$And_Value(GWAS:PUBMEDID)=FILTER(?V0 IN ("18846501"^^type:pubmed,"23535732"^^type:pubmed))

A final query $And_Union is constructed for all chosen facets F_1,F_2,...,F_m as

```
$And_Union=
?subject F_0 ?V0
$And_Value(F_0)
?subject F_1 ?V1
$And_Value(F_1)
....
....
?subject F_m ?Vm
$And_Value(F_m)
```

An example is

```
$And_Union=
?subject HBV:SAMPLE ?V0
FILTER(?V0 IN ("23","71"))
?subject HBV:TISSUE ?V1
FILTER(?V1 IN ("N","T"))
```

Construct General Query

The final step is to setup $Key_Query variable as follows.
$Key_Query_User contains the query entered through the SPARQL editor, or is empty otherwise.
$Query_String contains the text for free-text search.
$Limit is the number of hits to return.

```
if($Query_String)
{
	$OR="FILTER ($OR_Union_Text)" if($OR_Union_Text);
	$Wild_Predicate="?subject ?p ?o ." if($OR_Union_Text || $And_Union);
	if(!$Key_Query_User)
	{
	$Key_Query=
		"
			SELECT DISTINCT ?subject ?score
			WHERE
			{
				(?subject ?score) text:query ('$Query_String' $Search_Limit).
				$Wild_Predicate
				$And_Union
				$OR
			}
		";
	}
	else
	{
	$Key_Query=
```

```
				"
				SELECT DISTINCT ?subject ?score
				WHERE
				{
					$Key_Query_User
					?subject ?p ?o .
					$And_Union
					$OR
				}
				$Limit
			";
		}
	}
	else
	{
		$OR_Str="";

		if($OR_Union_Text)
		{
				$OR_Str="FILTER ($OR_Union_Text)";
		}
		if($OR_Union_Text || $And_Union)
		{
			if($Key_Query_User)
			{
				$Key_Query=
					"
					SELECT DISTINCT ?subject
					WHERE
					{
						$Key_Query_User
						?subject ?p ?o .
						$And_Union
						$OR_Str
					}
				$Limit
			}
			else
			{
				if(!$And_Union)
				{
					$Key_Query=
						"
						SELECT DISTINCT ?subject
						WHERE
						{
							$OR_Union
						}
```

```
					$Limit
				}
				else
				{
					$Key_Query=
						"
						SELECT DISTINCT ?subject
						WHERE
						{
							?subject ?p ?o .
							$And_Union
							$OR_Str
						}
					$Limit
				}
			}
		}
		else
		{
			$Key_Query=$Key_Query_User;
		}
	}
```
