# Supplementary material for: BioCarian: search engine for exploratory searches in heterogeneous biological databases
Source: BMC Bioinformatics. 2017 Oct 2;18:435. doi: 10.1186/s12859-017-1840-4 (PMC5625622; doi:10.1186/s12859-017-1840-4)
Supplement: Supplementary file 2 — Survey Detailed Results. (PDF 656 kb) [file 12859_2017_1840_MOESM2_ESM.pdf]

User Survey Detailed Results

Rate the applications based on their user friendliness.

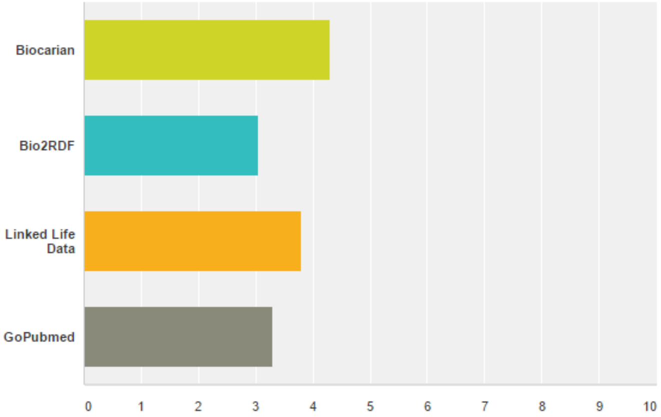

	Bad		Average		Excellent	Weighted Average
**Biocarian**	**0.00%** 0	**0.00%** 0	**20.00%** 4	**30.00%** 6	**50.00%** 10	4.30
**Bio2RDF**	**10.00%** 2	**15.00%** 3	**55.00%** 11	**0.00%** 0	**20.00%** 4	3.05
**Linked Life Data**	**5.00%** 1	**0.00%** 0	**35.00%** 7	**30.00%** 6	**30.00%** 6	3.80
**GoPubmed**	**10.00%** 2	**5.00%** 1	**45.00%** 9	**25.00%** 5	**15.00%** 3	3.30

Rate the applications based on their design

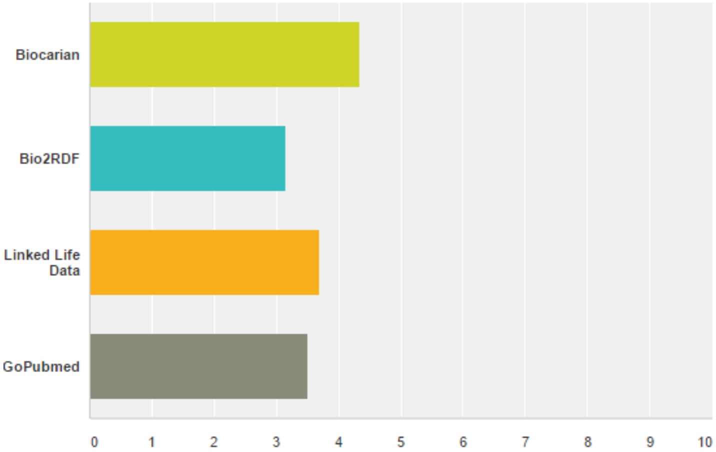

	Bad		Average		Excellent	Weighted Average
**Biocarian**	**0.00%** 0	**5.00%** 1	**15.00%** 3	**20.00%** 4	**60.00%** 12	4.35
**Bio2RDF**	**15.00%** 3	**20.00%** 4	**25.00%** 5	**15.00%** 3	**25.00%** 5	3.15
**Linked Life Data**	**5.00%** 1	**5.00%** 1	**25.00%** 5	**45.00%** 9	**20.00%** 4	3.70
**GoPubmed**	**5.00%** 1	**15.00%** 3	**30.00%** 6	**25.00%** 5	**25.00%** 5	3.50

Rank the applications on the ease to navigate facets

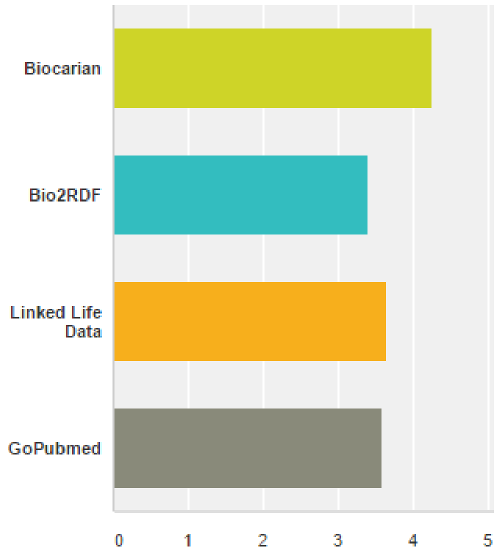

	Bad		Average		Excellent	Weighted Average
Biocarian	0.00% 0	5.00% 1	15.00% 3	30.00% 6	50.00% 10	4.25
Bio2RDF	15.00% 3	10.00% 2	30.00% 6	10.00% 2	35.00% 7	3.40
Linked Life Data	0.00% 0	15.00% 3	35.00% 7	20.00% 4	30.00% 6	3.65
GoPubmed	5.00% 1	5.00% 1	35.00% 7	35.00% 7	20.00% 4	3.60

In your opinion, the number of facets shown are:

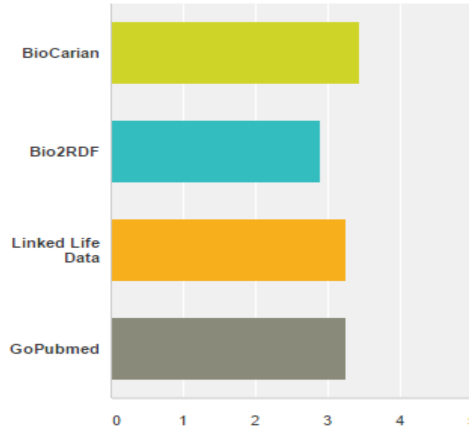

	Insufficient		Just the right Amount		Too much	Weighted Average
BioCarian	0.00% 0	0.00% 0	70.00% 14	15.00% 3	15.00% 3	3.45
Bio2RDF	20.00% 4	20.00% 4	30.00% 6	10.00% 2	20.00% 4	2.90
Linked Life Data	0.00% 0	25.00% 5	40.00% 8	20.00% 4	15.00% 3	3.25
GoPubmed	0.00% 0	20.00% 4	50.00% 10	15.00% 3	15.00% 3	3.25

Rank the applications by the methods they provide users to organize facets.

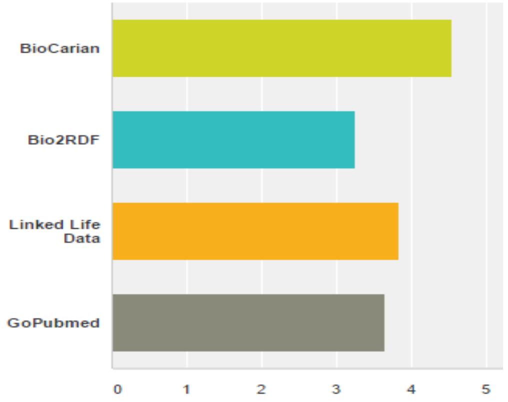

	Bad		Average		Excellent	Weighted Average
BioCarian	0.00% 0	0.00% 0	15.00% 3	15.00% 3	70.00% 14	4.55
Bio2RDF	10.00% 2	15.00% 3	40.00% 8	10.00% 2	25.00% 5	3.25
Linked Life Data	0.00% 0	5.00% 1	35.00% 7	30.00% 6	30.00% 6	3.85
GoPubmed	5.00% 1	10.00% 2	30.00% 6	25.00% 5	30.00% 6	3.65

Rank the following features in BioCarian according to their usefulness

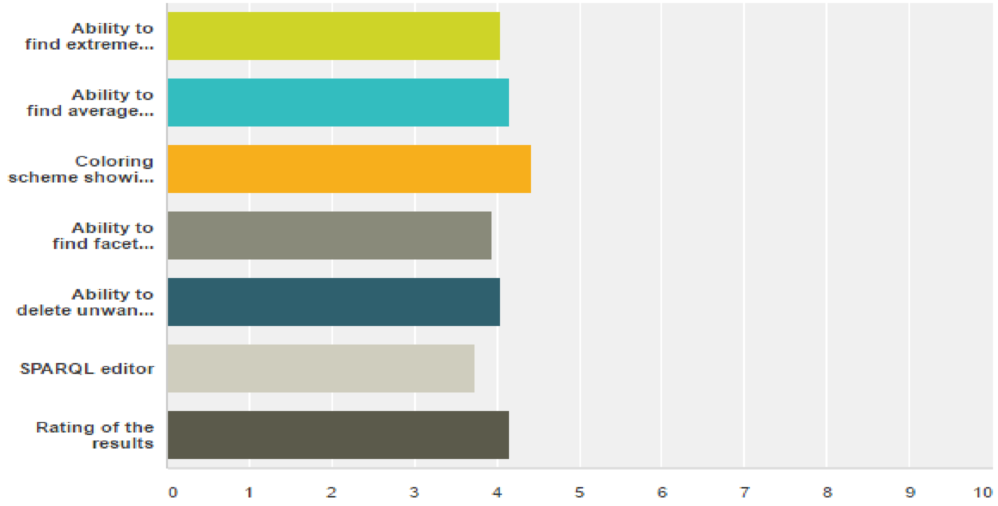

	not useful		useful		very useful	Total	Weighted Average
Ability to find extreme facet values	0.00% 0	10.00% 2	30.00% 6	5.00% 1	55.00% 11	20	4.05
Ability to find average or "normal" facet values	0.00% 0	0.00% 0	35.00% 7	15.00% 3	50.00% 10	20	4.15
Coloring scheme showing how far a facet value deviates from normal	0.00% 0	0.00% 0	21.05% 4	15.79% 3	63.16% 12	19	4.42
Ability to find facet values most relevant to current search	0.00% 0	0.00% 0	45.00% 9	15.00% 3	40.00% 8	20	3.95
Ability to delete unwanted facets from the view	0.00% 0	0.00% 0	35.00% 7	25.00% 5	40.00% 8	20	4.05
SPARQL editor	0.00% 0	10.53% 2	42.11% 8	10.53% 2	36.84% 7	19	3.74
Rating of the results	0.00% 0	0.00% 0	26.32% 5	31.58% 6	42.11% 8	19	4.16
